# Degradation of Polylactic Acid/Polypropylene Carbonate Films in Soil and Phosphate Buffer and Their Potential Usefulness in Agriculture and Agrochemistry

**DOI:** 10.3390/ijms25010653

**Published:** 2024-01-04

**Authors:** Izabela Szymanek, Martin Cvek, Diana Rogacz, Arkadiusz Żarski, Kamila Lewicka, Vladimir Sedlarik, Piotr Rychter

**Affiliations:** 1 Faculty of Science and Technology, Jan Dlugosz University in Czestochowa, 13/15 Armii Krajowej Av., 42-200 Czestochowa, Poland; izabela.szymanek@doktorant.ujd.edu.pl (I.S.); d.rogacz@ujd.edu.pl (D.R.); arkadiusz.zarski@ujd.edu.pl (A.Ż.); k.lewicka@ujd.edu.pl (K.L.); 2 Centre of Polymer Systems, University Institute, Tomas Bata University in Zlín, Trida T. Bati 5678, 760 01 Zlín, Czech Republic; cvek@utb.cz (M.C.); sedlarik@utb.cz (V.S.)

**Keywords:** biodegradable polymers, polypropylene carbonate, polylactide, polymer ecotoxicity, agrochemistry

## Abstract

Blends of poly(lactic acid) (PLA) with poly(propylene carbonate) (PPC) are currently in the phase of intensive study due to their promising properties and environmentally friendly features. Intensive study and further commercialization of PPC-based polymers or their blends, as usual, will soon face the problem of their waste occurring in the environment, including soil. For this reason, it is worth comprehensively studying the degradation rate of these polymers over a long period of time in soil and, for comparison, in phosphate buffer to understand the difference in this process and evaluate the potential application of such materials toward agrochemical and agricultural purposes. The degradation rate of the samples was generally accompanied by weight loss and a decrease in molecular weight, which was facilitated by the presence of PPC. The incubation of the samples in the aqueous media yielded greater surface erosions compared to the degradation in soil, which was attributed to the leaching of the low molecular degradation species out of the foils. The phytotoxicity study confirmed the no toxic impact of the PPC on tested plants, indicating it as a “green” material, which is crucial information for further, more comprehensive study of this polymer toward any type of sustainable application.

## 1. Introduction

Polymers have become an integral part of everyday life. This has, however, serious consequences, especially for the environment [1]. The pollution caused by plastics is a major aspect that has emerged in research on biodegradable polymers [2,3]. Currently, intensive development and application of biodegradable polymers into various fields, such as agriculture [4], industry [5,6], food [7,8], chemistry [2,9], medicine and related departments [10,11], and even boatbuilding [12], have been observed. However, unfortunately, this tendency does not allow for the elimination of classical non-biodegradable polymers. For many years, polyolefins have been widely used due to their mechanical properties, including durability, impermeability, strength, and price, and unfortunately, they still represent the vast majority of polymer consumption in all industrial branches all over the world, including food packaging and agriculture [13,14]. Searching for new environmentally friendly polymers that allow for the reduction in greenhouse gas emissions or landfill site numbers is still a global challenge due to the enormous plastic waste generated after use, including microplastics in the air, aquatic reservoirs, and soil. For this reason, environmentally friendly polymers should be an alternative to conventional plastic wherever it is possible. One of the most popular and very-well-studied biopolymers is polylactide (PLA), which has found an application in many areas of our daily lives due to its mechanical and other useful properties similar to those of traditional poly(ethylene terephthalate) (PET) or polyethylene (PE) [15]. However, despite its good availability, relatively low price, and useful properties, PLA still has many disadvantages that limit its use. For example, although the hardness of PLA is higher than that of polystyrene (PS), it still cannot compete with PET and polycarbonate PC, which can consequently limit the use of PLA as a construction material. Also, the biomedical applications of PLA are not very common due to its slow biodegradation and hydrophobicity [16]. In recent years, polypropylene carbonate (PPC) has become one of the most sought-after biodegradable polymers. This polymer is considered a green polymer due to the fixation of CO_2_ during the polymerization process, leading to a reduction in greenhouse gases [17]. Its thermal decomposition is between 150 and 180 °C, and the resulting product is mostly cyclic propylene carbonate [18]. This may be a disadvantageous phenomenon because, during processing at high temperatures, high stresses may occur, and after cooling, deformations may take place. However, there are various methods to improve the thermal stability of PPC, e.g., modifying the backbone with ether units. The glass transition temperature (*T*_g_) ranges from 25 to 45 °C depending on the structure of the main chain and molecular weight. It can also be regulated with plasticizers [18,19,20]. At higher temperatures, PPC exhibits the behavior characteristic of a pseudoplastic, non-Newtonian fluid. It was also shown that the tensile strength of this polymer is 21.5 MPa, Young’s modulus is 330 MPa, and elongation at break is 330% [18]. However, its compressive strength is low (100–300 kPa) [19]. Thanks to these parameters, PPC can be used as an additive for plastics in the food packaging industry. By adding PPC to other materials, such as starch or poly(buthylene adipate-co-terepthalate) (PBAT), its mechanical parameters can be effectively modulated. Initially, immediately after its discovery in the 1920s, it was used as a binder [18,19,20]. The combination of PPC with nanocellulose increased the tensile strength of the resulting material, as well as its elongation at break and temperature stability [21]. It also shows better impermeability to water vapor than PLA; therefore, it can be used to create blends with other polymers and then used in this form in the food industry [8].

Since PLA is one of the most commonly biodegradable polymers used for blend preparation with other biodegradable polymers like polycaprolactone, polybutylene succinate, poly(butylene adipate-co-terephthalate), or polyhydroxyalkanoates (PHAs) [16,22], we aimed to evaluate the degradation behavior of PLA/PPC blends (in various ratios) in soil and phosphate-buffered saline (PBS). The obtained results should point out the possibility of using such blends for agriculture purposes as biodegradable mulching films or in agrochemistry for the preparation of slow-release active agents like herbicides or fertilizers using PLA/PPC blends as a polymer carrier. Moreover, according to our knowledge, there are no reports devoted to the degradation of PPC and its blend in soil over a 2-year period, nor are there any phytotoxicological studies of PPC. The preliminary results demonstrated that the degradation rate can be altered by changing the composition of PLA/PPC blends. This is especially important from a plant cultivation point of view because the release of an active agent will undoubtedly be determined by the PPC/PLA composition.

The existing literature has reported on PLA/PPC blends to indicate their potential for future applications. For example, PLA has been successfully blended with PPC using melt compounding [23] and modified with plasticizers such as maleic anhydride, yielding a noticeable increase in elongation at break dependent on the PPC content. Using differential scanning calorimetry (DSC) analysis, it was reported that there are two different glass transition temperatures. The shifting of the *T*_g_ of PLA to a lower value and vice versa, with an increasing amount of PPC, indicated the partial miscibility of PLA and PPC. This finding was further confirmed by microstructural analysis, which revealed a uniform distribution of both components. Moreover, infrared spectroscopy showed possible intermolecular interactions between the -CH group of PLA and the -O-C or -O=C PPC groups, indicating the partial miscibility of the blend. PPC had a positive impact on the barrier characteristics of the PLA/PPC blends, including water vapor and oxygen permeability [24,25].

Considering recent studies on the application of PLA/PPC blends in the sustainable packaging industry, there is an urgent need to investigate the (bio)degradation process of both PPC itself and its blends with PLA, both in the soil and in the aqueous environment, because a lot of plastic packaging wastes pollute the soil environment due to improper practices by consumers or inadequate waste management. In this study, two types of PLA (crystalline and amorphous) were used for the blend preparation with PPC in order to compare their degradation rates in both tested environments. Based on the degradation profile obtained, it will be easier to design their composition for agriculture and agrochemical applications. The same blends were used in the degradation study in PBS buffer for comparison.

## 2. Results

### 2.1. Weight Changes in Polymer Samples

Based on previous reports, it is known that PLA degrades enzymatically in both soil and water [26,27]. This is the reason why PLA is often blended with other polymers that are more susceptible to microbial attack, like, for example, polyhydroxybutyrate (PHB) or PBAT [28]. Since there are no reports related to the degradation of PLA/PPC blends and PPC in soil in a 24-month period and only scarce information about PLA/PPC degradation in PBS exists, there is a need to study the behavior of these materials in the mentioned media [17]. This is especially important in view of agriculture and agrochemistry. The rate of degradation may drive a further potential application of these blends toward the fabrication of mulching films or control release systems of agrochemicals using these blends as a polymer carrier (slow-release fertilizers or pesticides). The PBS solution was selected as a medium because it better simulates ground water compared to distilled water and is often used for in vitro degradation studies of aliphatic biodegradable polyesters [29].

#### 2.1.1. Weight Loss of Sample in Soil

**Figure 1:**
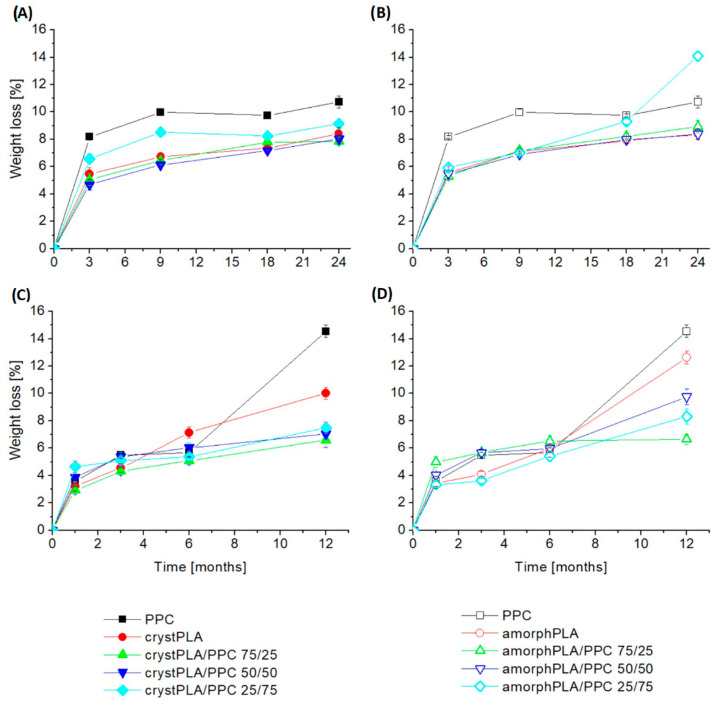
Weight loss of polymers and blends during incubation in soil and phosphate buffer. (**A**,**B**)—incubation in soil, (**C**,**D**)—incubation in buffer.

The results of the weight loss during the degradation process of polymers and their blends are presented in Figure 1. In general, pure PPC was found to demonstrate the highest weight loss, and its contribution to the blends enhanced their weight loss. The greatest weight loss occurred for PPC foils and blends with their predominance. Until the 9th month, the lowest weight values were observed for the PPC sample (8.2% after 3 months and 10% after 9 months), then for the crystPLA/PPC 25/75 blend (6.6% after 3 months and 8.5% after 9 months), and in third place, amorphPLA/PPC 25/75 (6% after 3 months and 7% after 9 months). However, in the following months, a change occurred, which resulted in the blend with amorphous PLA having the second highest weight loss reading. Finally, after 24 months, the samples with the greatest weight loss can be ranked as follows: amorphPLA/PPC25/75 > PPC > crystPLA/PPC25/75 with the following results: 14%, 10.7%, and 9.1% of weight loss. Among the pure films, smaller weight losses were observed for crystalline PLA than for amorphous PLA. The biggest one is for PPC. Among the blends containing crystalline PLA, the largest weight loss was recorded for the blend with a predominance of carbonate, and the smallest for the crystPLA/PPC 50/50 sample. However, after 24 months, the smallest weight loss occurred for the sample with a predominance of PLA. In the case of blends containing amorphous PLA, the results were comparable up to the 6th month of incubation. Later, however, it could be observed that the largest weight loss occurred for the sample with a predominance of PPC, and the smallest for the amorphPLA/PPC 50/50 blend. It is also worth noting that blends containing amorphPLA were characterized by greater mass loss than their counterparts containing crystPLA in the final phase of the study.

#### 2.1.2. Weight Loss of Samples in PBS

In the case of samples incubated in PBS, among pure polymer films, the fastest weight loss occurred for PPC (after 12 months, 14.5% weight loss), and the slowest, up to 6 months, for amorphous amorphPLA; however, after a year, the smallest weight loss was recorded for crystalline crystPLA (10%). For blends containing crystalline PLA, the smallest weight loss occurred for the crystPLA/PPC 75/25 blend, and this tendency persisted throughout the entire incubation period. However, after the first month and after a year of incubation, the largest loss occurred for the PPC-dominant foil, and after 3 and 6 months for the crystPLA/PPC 50/50 blend. Among the blends containing amorphous PLA, up to the 6th month of incubation, the largest weight losses occurred for the amorphPLA/PPC 75/25 sample, and the smallest for the 25/75 ratio. After a year, however, the largest losses occurred for the 50/50 blend, and the smallest for the amorphPLA/PPC 75/25 sample. When testing blends with the same ratio of PLA to PPC, it can be noticed that, as in the case of blends incubated in soil, samples containing amorphous PLA show a greater weight loss than their crystalline counterparts. Finally, after a year, the films made of pure polymers were characterized by the greatest weight loss.

Comparing the results for both incubation environments, it can be noticed that in both cases, the highest weight losses were recorded for the PPC foil. In the case of samples incubated in soil, the results are similar, while the results of samples incubated in PBS are further apart.

The degradation process was accompanied by a gradual decrease in pH values, regardless of the medium. In comparison, the pH values of all blends immersed in PBS were lower than those in the soil. Specifically, pH reached the lowest values of 4.9 for amorphPLA after 12 months in PBS and 5.5 after 12 months in soil. An increasing content of PLA in the blends yielded higher acidity in the medium. This is due to the release of low molecular degradation products of PLA, including lactic acid. The impact on the acidity was more pronounced for the amorphPLA grade compared to the crystPLA.

### 2.2. Changes in Molecular Weight of Incubated Samples

#### 2.2.1. Degradation of Samples in Soil

**Figure 2:**
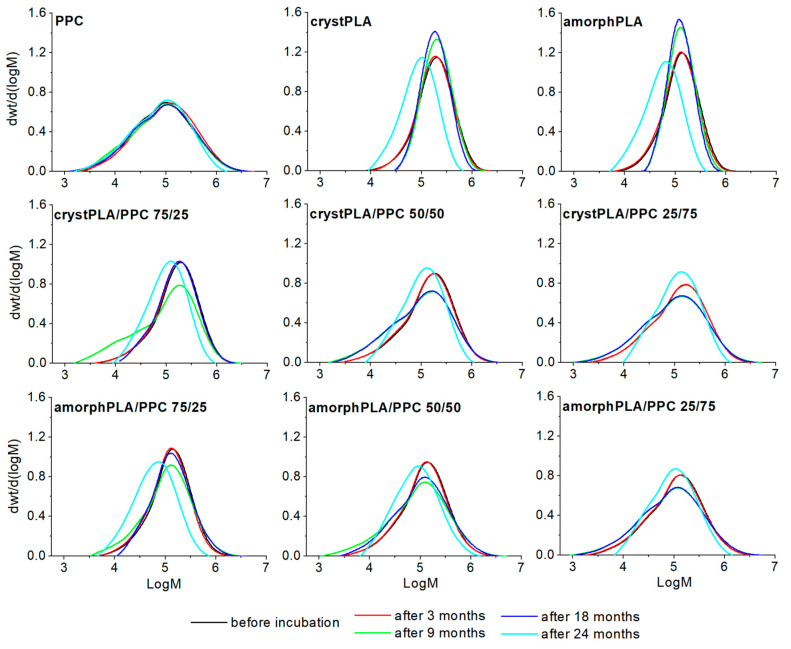
GPC elugrams of examined polymers incubated in soil.

Knowledge of the degradation process of pure materials (PLA and PPC) and their blends, both in the soil and in the PBS environment, is crucial in assessing the impact of these polymers on the environment. Gel permeation chromatography (GPC) is the fundamental source of information about the degradation process caused by the breakage of polymer main chains [30]. The results of the GPC analysis (Figure 2, Table 1) indicate that progressive hydrolytic degradation was occurring in the soil environment for all the tested polymer blends. While no significant changes in molecular weight were recorded in the first period of the study (3 months), a significant decrease in the number-average molar mass (Mn) was detected after 9 months of incubation in the soil. At the same time, a significant increase in the polydispersity index (*Đ*) was noted compared to the value of the starting polymers. There was a significant reduction in both the Mn and the weight-average molar mass (Mw) for the PPC reference. After 9 months of incubation in soil, Mw decreased by 9%, and after 24 months by 20%. The *Đ* value for this sample also decreased (from 5.28 to 4.54), which indicates the penetration of low molecular degradation products into the soil. For crystPLA, a small increase in Mn can be observed within 9 and 18 months of incubation in soil, but after 24 months, this value drops by half. Such changes, especially the increase in Mn, are a known phenomenon and usually indicate the leaching of low molecular decomposition products from the sample into the surroundings, which causes a temporary increase in molecular weight. This is confirmed by elugrams, which show a shift of the peaks towards lower molecular weights after 24 months. The relatively symmetrical shape of the elugrams indicates the uniform nature of the decrease in molecular weight throughout the degradation period. This is also confirmed by the *Đ* value, which remains almost constant throughout the incubation period. A similar situation was observed in the case of amorphPLA. As expected, the molecular weight after 24 months was much lower than in the case of crystPLA, whose crystalline regions represented a barrier for the penetrating water molecules [31].

In the case of crystPLA/PPC blends, after 3 months of incubation in the soil, minimal changes in molecular weights were recorded; however, a significant decrease in the Mn value was visible after 9 months (75/25 > 50/50 > 25/75 by 64, 44, and 34%, respectively), which was accompanied by a doubling of the *Đ* value. After 18 months of degradation in the soil, a similar trend was revealed; however, both the Mn values and the nature of the elugram curves (shifting towards higher masses) of blends containing the highest PLA content (both crystalline and amorphous) indicate the leaching of low molecular degradation products of these blends into the soil, which, as a consequence, results in an increase in Mn and a lower *Đ*. The same type of degradation was observed for crystPLA and amorphPLA/PPC blends after 24 months. This means that in these blends (with a higher content of PPC), the washing out of the degradation products took longer than in the case of high amounts of PLA in the blend. This confirms the nature of elugram curves with essential tails toward low Mn (after 18 months) and no tail and shift toward a higher molecular weight (after 24 months). Comparing blends of PPC with amorphous and crystalline PLA, as expected, the first one degrades faster due to its higher diffusivity for water in contrast to the hindrance of water caused by the crystalline regions of crystPLA.

#### 2.2.2. Degradation of Samples in PBS

Analysis of the Mn, Mw, and *Đ* values and the nature of the elugram curves (Table 2, Figure S1) indicates a gradual decrease in the molecular weight of PPC. In comparison, both amorphous and crystalline PLA degrade in a much more unpredictable way when compared to their degradation profiles in soil. Hydrolysis of the PLA foil can take place as a result of surface erosion, throughout the entire volume of the material, or erosion with accelerated degradation of the core. The contributions of individual mechanisms depend on the sample morphology, the composition of the hydrolyzing medium, and the temperature. A high hydrolysis rate (compared to the rate of diffusion of the hydrolyzing medium into the sample) leads to surface erosion, and a low hydrolysis rate leads to erosion throughout the sample volume [32]. If the products of hydrolytic decomposition of PLA are released very slowly from deep within the material and at the same time increase the rate of hydrolysis (autocatalytic effect), then accelerated erosion of the sample core occurs. Partially crystPLA hydrolyzes much faster in amorphous areas. During the release of degraded PLA chains, the additional crystallization of amorphous PLA occurs [33,34]. After a year of incubation in PBS buffer, the Mn of these polymers decreased dramatically (Table 2), which indicates the almost the complete degradation of these polymers in the environment. It is worth noting a certain tendency here: among the blends, the higher the content of PPC, the lower the decrease in Mn was. Let us emphasize that the blend with amorphPLA had a much higher decrease in molecular weight (almost twice after a year of incubation). This is confirmed by increasing the *Đ* and elugram curves of GPC in PBS.

### 2.3. Changes in Blends Compositions

**Figure 3:**
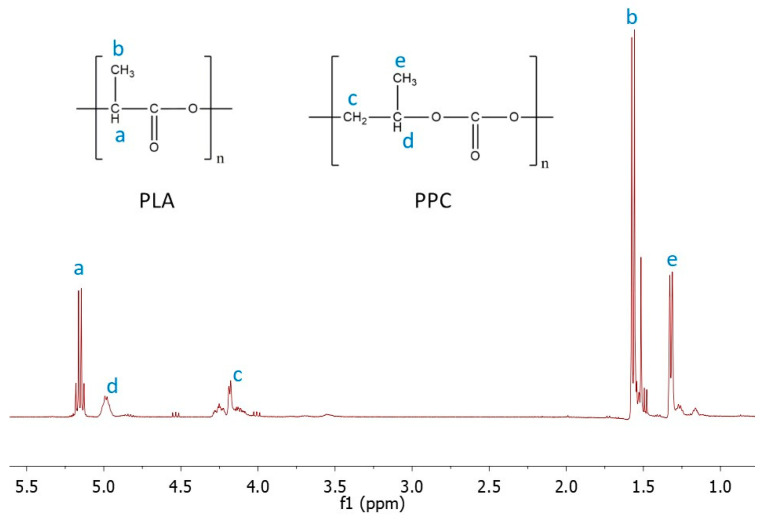
^1^H NMR spectrum of PLA/PPC blends with signals assigned to the appropriate protons: a, b correspond to proton signals assigned to -CH- and -CH_3_ groups of PLA, respectively, and c–e correspond to proton signals assigned to -CH_2_-, -CH- and -CH_3_ groups of PPC, respectively.

Nuclear magnetic resonance (NMR) is a very useful technique for the determination of polymeric blend composition [26,28,35]. This method successfully helps to calculate the ratio of both components, indicating if some of them are more privileged for degradation in the tested medium. The representative ^1^H NMR spectrum of the PLA/PPC blend is displayed in Figure 3, and a summary of the results is presented in Table 3. Results related to blend composition are ambiguous. Generally, changes in blend composition do not depend on the type and amount of PLA in the blends. It was expected that blends containing amorphPLA would allow for the faster release of PPC and the corresponding degradation products; however, based on the NMR analysis, the composition of the blends was comparable in both tested media during degradation time. Amorphous PLA did not facilitate water penetration into the blend film, resulting in faster polymer degradation. Composition changes in the soil and PBS were comparable in time regardless of the amount of PPC in the blends.

### 2.4. Changes in Blends Crystallinity

Differential scanning calorimetry (DSC) is one of the main thermal analysis techniques. By examining various thermodynamic processes, phase transitions, specific heat capacity, and related kinetic properties, it can be used to explain the structural changes and crystallinity of polymers [36]. DSC can also be used to evaluate the degradability of these compounds under the influence of various factors, both biotic and abiotic [37]. In the presented studies, DSC was carried out to determine the changes that occur in relation to phase transformations in pure polymers and their blends during progressive degradation in soil (Table 4) and PBS (Table S1). In general, the results confirmed the semi-crystalline or amorphous nature of the obtained blends depending on the qualitative and quantitative composition of the individual samples. Typical phase transitions characteristic of amorphous polymers (PPC, amorphous PLA), i.e., the glass transition, and crystalline thermoplastics (semi-crystalline PLA), i.e., in addition to the glass transition, an exotherm related to cold crystallization and a melting endotherm, can be observed. The DSC analysis also showed that the glass transition temperatures for semi-crystalline and amorphous PLA and PPC differed significantly depending on their composition as well as on the degradation conditions and exposure time. The *T*_g_ of pure PPC was approximately 11.8 °C, while for PLA it was 44.6 °C and 38.5 °C for the semi-crystalline and amorphous varieties, respectively. In the PLA/PPC blends not subjected to degradation, the *T*_g_ values for PLA were lower and, in the case of PPC, higher compared to the values before the blend was obtained. Based on the analysis of cold crystallization temperatures (*T*_cc_), and mainly crystallization and melting enthalpies, it was examined how the rate of possible crystal nucleation and the degree of crystallinity of pure samples and their mixtures changed depending on the degradation progress. The general tendency was that with the longer degradation time, the *T*_cc_ of PLA increased and the enthalpy of the process decreased, which indicated a lower nucleation ability and a less intense crystallization process, respectively.

### 2.5. Surface Films Erosion

The observation of changes in the surface of the tested samples is a useful method for the initial assessment of the advancement of the degradation process. When visually assessing samples, a change in the color of the sample surface is initially visible, which may be caused by microorganisms present on the sample surface. The color change in the sample is the first stage of degradation, called biodeterioration. These changes contribute to the deterioration of the material’s properties. The next stages of biodegradation are biofragmentation and assimilation. When observing the polymeric films with scanning electron microscopy, the appearance of holes on the surface of the sample is visible, which ultimately leads to their disintegration [38,39].

**Figure 4:**
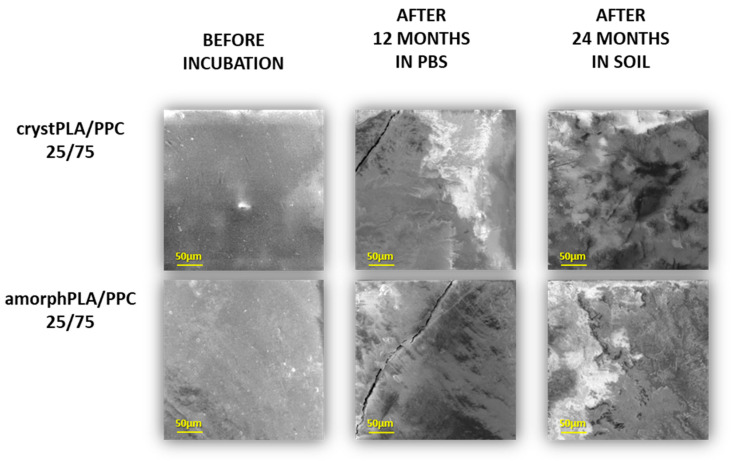
The selected SEM micrographs of the surfaces of incubated foils before and during incubation.

Figure 4 shows the most representative changes in the surface erosion of samples of degradation after 12 months in PBS and 24 months in soil. At the start of degradation, the surface of polymeric discs was quite smooth and nonporous; however, along with progress in time, we observed progress in surface degradation that was more and more visible as irregular defects such as splits and cracks. Among pure polymers, the highest surface erosion after incubation in soil can be observed for pure PPC (Figure S2). The erosion of the PLA film is visible to a greater extent for the amorphPLA than for the crystPLA. However, blends of samples with a predominance of PLA showed a higher degree of surface erosion than both pure PLA samples. Particular defects in the surface are visible for pure PPC and blends (crystPLA/PPC 25/75, amorphPLA/PPC 25/75), which contain a larger content of PPC. Polymeric samples immersed in PBS mostly undergo hydrolytic degradation, which is possible due to the penetration of water molecules into the polymeric matrix and the cleavage of bonds in polymer chains. This type of degradation has less impact on the surface of the sample. In the case of soil, the activity of microorganisms considerably increased, and therefore, enzymatic degradation resulted in surface erosion. In relation to this, two types of degradation processes in the tested polymeric samples have been observed. In the first, which is hydrolytic degradation, the bonds of the main polymer chains are cleaved, thereby reducing the size of the polymer chain. The second, which is enzymatic degradation, occurs via surface erosion, where microorganisms start consuming polymers enzymatically from the surface, thereby causing a significant decrease in the weight of the polymeric sample.

### 2.6. Plant Growth Tests

**Figure 5:**
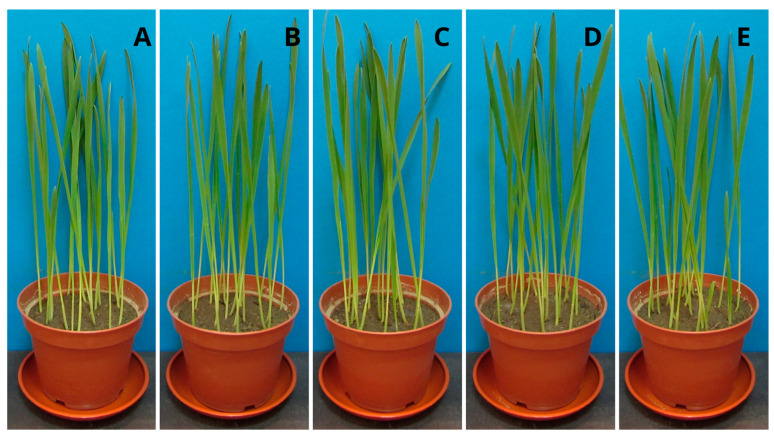
Oat seedlings (*Avena sativa*): (**A**) control sample (without PPC), (**B**) 250 mg, (**C**) 500 mg, (**D**) 750 mg, and (**E**) 1000 mg of PPC/kg soil dry matter.

**Figure 6:**
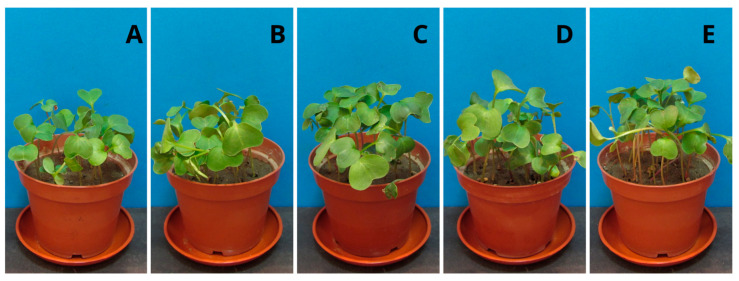
Radish seedlings (*Raphanus sativus*): (**A**) control sample (without PPC), (**B**) 250 mg, (**C**) 500 mg, (**D**) 750 mg, and (**E**) 1000 mg of PPC/kg soil dry matter.

**Figure 7:**
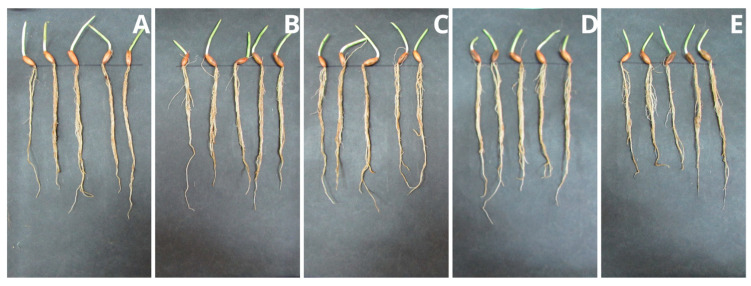
Root systems of oat seedlings (*A. sativa*): (**A**) control sample (without PPC), (**B**) 250 mg, (**C**) 500 mg, (**D**) 750 mg, and (**E**) 1000 mg of PPC/kg soil dry matter.

**Figure 8:**
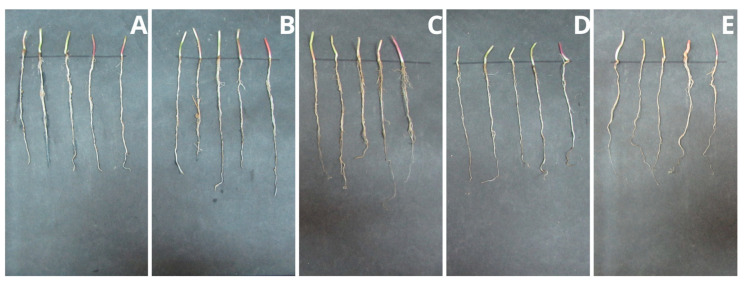
Root systems of radish seedlings (*R. sativus*): (**A**) control sample (without PPC), (**B**) 250 mg, (**C**) 500 mg, (**D**) 750 mg, and (**E**) 1000 mg of PPC/kg soil dry matter.

**Figure 9:**
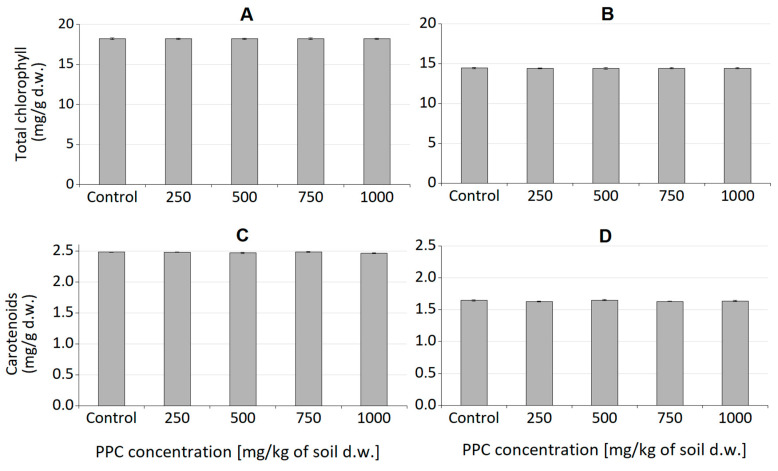
(**A**,**B**)—Chlorophyll content in oat and radish seedlings, respectively. (**C**,**D**)—carotenoids content in oat and radish seedlings, respectively [mg/g of dry weight]. Error bars represent standard deviation of three replicates.

Studying the impact of the polymer on plants is extremely important due to its potentially industrial nature. Higher plants, as the first creatures exposed to potentially harmful PPC decomposition products, can indicate the dangers of the material entering the environment. The height of the seedlings of the tested samples with the addition of PPC and control samples (Figure 5 and Figure 6), their root systems (Figure 7 and Figure 8), and assimilation pigments (Figure 9) was compared.

In the case of oat, the results of germination (Table 5) and fresh weight (Table 6), regardless of the concentration of PPC used, were comparable to those of the control plant. Increasing the concentration of PPC in the soil reduced the root length and dry mass of oat seedlings. In the case of radish, there was an increase in the fresh and dry weight of the plant at each concentration of PPC in the soil.

The difference in the effects that the test substances have on the plant also depends on the type of plant. Oats belong to the group of monocotyledonous plants, while radish is a dicotyledonous plant. These plants have different root systems and a different water transport system. Therefore, their reaction to a given substance may be different. Comparing these plants, oat is found to be more sensitive when treated with increasing concentrations of PPC.

Fresh mass and root length were selected parameters of both plants growth for the determination of the NOEC and LOEC (No Observed Effect Concentration and the Lowest Observed Effect Concentration) values. Due to the negligible toxicity on the fresh mass of both of the tested plants (the magnitude of the biological effect is 10 to 30%), it was impossible to calculate these values. However, in the case of root length, very low toxicity against oat allowed for the determination of NOEC and LOEC values (750 mg and 1000 mg, respectively). Radish roots were found to be persistent in the presence of PPC.

The results obtained from the program GraphPad demonstrated an EC_50_ value (half maximal effective concentration) only for oat roots, and it amounted to 2796 mg/kg of soil d.w. With this respect, tested polymers may be considered non-toxic materials for plants.

Chlorophyll plays a crucial role in photosynthesis and supports plant growth, and, together with carotenoids, it is a sensitive indicator of the damaging impact of various biotic and abiotic stresses on leaf function and health [40,41]. Analysis of the level of assimilation dyes treated with PPC in the soil did not show a significant effect, regardless of the concentration introduced into the soil. Both chlorophyll and carotenoids remained at levels comparable to those of control plants. This means that this polymer does not negatively affect plant photosynthetic pigments. The obtained results coincide with the phytotoxicological study of PLA films, which we already reported [26]. Plant growth tests performed with cress (*Lepidium sativum*) and barley (*Hordeum vulgare*) confirmed that PLA and its degradation products were completely non-toxic to the tested plants. The same is true for natural polyhydroxybutyrate and its atactic and amorphous analogue, amorphPHB. With this respect, PPC can be successfully used for agricultural and agrochemical purposes. Obviously, more comprehensive studies are still required; however, for the first time, according to our knowledge, a phytotoxicological study of PPC was conducted.

## 3. Discussion

Due to the long-term degradation of PLA, many attempts have been made over the years to accelerate this process by creating blends of PLA and other polymers. In the study by Naranćić et al. [42], it was shown that the weight loss of PLA/PCL blends was faster than PLA alone, while PLA mixtures with PBS, PHB, and polyhydroxyoctanoate (PHO) decomposed much slower. This experiment was performed in compost, so the results cannot be clearly compared with the present study. However, in the case of PHB/PLLA blends [26], films consisting of two polymers also decomposed faster than those containing only polylactide. A similar situation occurred in the study by Han et al. [43], where PLA/poly(3-hydroxybutyrate-co-4-hydroxybutyrate) (P(3HB-co-4HB)) blends were degraded in a PBS with the addition of *Amycolatopsis orientalis* ssp. *orientalis*. It was also noticed there that the blends degrade much faster than a foil consisting only of PLA. Copolymerization can also be a means of accelerating the degradation of polymers. According to research by Rychter et al. [44], the new poly(L-lactide-co-glycolide)-poly(ethyleneglycol)-poly(L-lactide-co-glycolide) (PLGA-PEG-PLGA) also showed a higher degree of degradation than its components. This was mainly thanks to PEG chains. Even the smallest addition significantly accelerated the surface erosion and weight loss of the foil. However, focusing on PPC degradation, the obtained results can be compared with the experiment [20]. There is a noticeable tendency for faster weight loss for PPC samples (in this case, a PPC/starch blend) incubated in PBS. The results for both experiments after a month are similar (approx. 3% loss for PLA and approx. 3.5% for PPC). This implies that PPC is more susceptible to hydrolytic and enzymatic degradation than PLA. However, polypropylene carbonate can also accelerate the degradation of blends with other materials. According to research by Pan et al. [45], PPC also accelerates the erosion of PBAT/PPC materials. In this case, as in the present study, increasing the percentage of PPC in the blend promoted a decrease in sample weight. There were losses of 25–30% after 3 months of incubation in the soil, so much more than in the above experiment. However, to further accelerate the degradation of PPC, Yang et al. [46] proposed adding tannic acid to this polymer. Thanks to this, it is possible to accelerate the weight loss of the polymer sample from approximately 6% (pure PPC) to up to 20% after 12 weeks of incubation in soil. It is worth noting that in this study, after 12 weeks, the weight loss of PPC itself was approximately 8%, which may be due to differences in soil composition and incubation conditions.

The pH readings presented in the study were analogous to the previous report [20], where similar results were achieved during incubation in PBS. There, also for samples containing PPC, the pH decreased less. This is the result of the decomposition of polylactide, one of the products of which is lactic acid, which acidifies the decomposition environment and lowers the pH. However, PPC, the decomposition products of which are propylene oxide and carbon dioxide [47], has a much smaller impact on the potential pH reduction in the environment in which the sample is located.

So far, there are no accurate results from measurements of the molar mass of PPC during degradation. However, it can be noted that in the case of thermal decomposition [48], the Mw parameter gradually decreases with increasing temperature while the *Đ* index increases, which is not so obvious in the case of degradation in the natural environment or PBS. The elugrams presented in this study also indicate the disintegration of long chains by temperature. However, according to the research of Di Lorenzo et al. [49], the PLA/PPC blends reduce the total molar mass, and these values differ significantly from the estimates. Nevertheless, these results are not consistent with the conducted experiment, which may be due to differences in the material (different PLA grade) and a different method of preparing the foil (mixing molten polymers). It is also possible to use poly(lactic acid)-b-poly(propylene carbonate) copolymers (PLA-b-PPC) using tetrabutyl titanate (TBT) as a catalyst [50]. However, in this study, the molecular weight of the samples decreased with the addition of TBT, which shows how much influence the catalyst has on the final product. Also, the *Đ* for these samples was much larger than in the present research. However, based on detailed information about the decomposition of PLA, certain dependencies can be noticed. Compared to the experiment by Araujo et al. [51], where, for films made of PLA and nanocomposites, a clear change in molecular weight can be observed after 6 weeks in compost at 40 °C; here, significant changes appear only after 9 months, which may depend on the degradation conditions. However, it is important to note that in both experiments, the elugram curves show a shift towards lower molecular weight, which indicates that the chain of long molecules was broken and shorter ones were formed. Similar conclusions were drawn from the work of Backes et al. [52], where, after composting PLA samples with the addition of algae, fragmentation of long polymer chains and their leaching into the sample environment were also demonstrated.

The degradation of pure PPC does not show changes in the polymer structure that can be observed using the NMR technique [53]. Unfortunately, there is no NMR data on PLA/PPC blends in the literature. The obtained results show, however, that PLA/PPC blends are characterized by a more uniform degradation process compared to Álvarez-Méndez et al. [35], where testing bags with a PLA/PBAT composition showed that the degree of polylactide loss after degradation is much greater than that of PBAT. These mixtures were not characterized by a uniform degree of decomposition. According to research [54] carried out on PLA/PET copolymers, a decrease in the PLA content can be observed with the incubation time. This means that the repeating PLA units covalently bonded to the repeating PET units in the copolymers can be decomposed by hydrolysis, thus showing a partial degradable character of these copolymers. However, in blends of biodegradable polymers, both components are distributed evenly, which may be important for their industrial use.

In the case of partially crystalline PLA and its blends with PPC, two melting endotherms were observed in the DSC curves both before and during degradation (Table 4 and Table S1). This effect is attributed to the formation of different crystalline states in the sample during subsequent thermal cycles [55,56]. Such polymorphs may exhibit different thermal stability, as is the case with α’ and the more stable α crystals [57]. The observed discrepancies and dispersion of *T*_g_ values compared to pure components indicate that PLA/PPC blends are only partially miscible, but due to their chemical nature, they remain compatible to some extent [23]. During the progressive degradation of the samples, even higher *T*_g_ values were recorded for polymers in mixed and unmixed forms. This may indicate that the subsequent degradation products obtained were not typically amorphous. The increase in the content of the crystalline phase in the hydrolysis residues could be manifested by an increase in the temperature of the transition to the glassy state. Gradual hydrolysis of the blends due to the formation of many short-chain products with increased molecular mobility should result in a gradual decrease in *T*_g_. However, throughout practically the entire period of degradation, it had quite high values. The semi-crystalline nature of the samples and, thus, their higher resistance to degradation may be due to the fact that these low-molecular-weight fractions may have the ability to recrystallize or self-nucleate [58]. Blends, especially after a longer incubation period during degradation, should show more similar *T*_g_ and lower ones compared to the material before incubation. The slow degradation may be caused not only by the polymorphism of PLA but also by the tendency of frequent reorganization of the amorphous fraction, which makes access to hydrolytic factors difficult. Partially crystalline PLA is known to hydrolyze much faster in amorphous areas. However, during the release of degraded PLA chains, the additional crystallization of amorphous PLA may occur, thus creating an additional obstacle to effective and rapid degradation [59]. When incubated at temperatures below *T*_g_, amorphous PLA fractions (and experiments assessing degradation were conducted under such conditions) are in a non-equilibrium state and tend towards greater order, i.e., equilibrium. This is the so-called isothermal or non-isothermal physical aging of PLA, during which relaxation processes result in the reversible ordering of the amorphous phase. This is another reason that may explain the hindering or slowing down of degradation processes in typically amorphous or semi-crystalline blends, which were the subjects of this research [60,61].

Previously conducted studies also show the ease of degradation of PPC surfaces [20]. In this experiment, both pure PPC and its blends with starch suffered significant surface damage. Similarly, in the case of the research by Jin et al. [62], PPC had one of the highest degrees of biodegradation among the tested polymers. Its surface erosion was visible to the naked eye after just 7 days of degradation in anaerobic conditions. This is confirmed by the results presented in our study, where erosion of the surface of PPC samples and blends containing a predominance of PPC is much more visible regardless of the incubation conditions. Similar results were obtained in the work of Pan et al. [45], where SEM photos clearly show that the higher the PPC content in the PBAT/PPC blend, the more damaged the foil surface. These damages were much larger than in the above experiment, which may be related to the nature of PBAT. Moreover, as found by Yang et al. [46], tannic acid can be added to further accelerate the erosion of the PPC foil surface, which yielded significant changes after a short incubation period (3 months). These were much bigger changes than in the case of pure PPC. However, research by Mistry et al. [63] showed that during the composting of PLA, its crystallinity may increase, which is manifested, among others, by the milky color of the foil. Such observations were also made in this study. Films containing polylactide became whiter after 1 month of incubation in the buffer. However, films containing a higher polylactide content are more resistant to degradation in soil, as also reported by [64], whose SEM photos of a PHA/PLA blend were also less damaged on the surface the more polylactide was in the sample. After three months of incubation, as in this study, the surface damage to the PLA-dominant foil was almost imperceptible.

As reported by Manzano et al. [65], PLA/PHB blends exerted no harmful effects on higher plants, and all parameters were comparable to the control. The tested polymers did not have a harmful effect on plants, unlike the PEG material [66], where an increase in the molecular weight and concentration of PEG in the soil resulted in a decrease in the content of chlorophyll and carotenoids in oat and radish leaves compared to the control plants. This means that PPC does not cause chlorosis or necrosis in plants that are exposed to its action. It is also possible to use some polymers as plant fertilizers. The study by Rychter et al. [67] showed that the nitrogen contained in polyethyleneimines stimulates plant growth (especially compounds with a linear structure). However, it is worth noting that branched polyethyleneimines adversely affect higher plants. Another material that can be used as a source of nitrogen in the soil is poly(methylene-co-cyanoguanidine) [68]. It did not have a harmful effect on the tested plants at low concentrations; however, at higher concentrations (2000 mg/kg of dry soil mass), the amount of nitrogen inhibited the development of oats and radishes. A similar relationship can be seen in the present study, where the highest concentrations of PPC had an adverse effect on the development of oat roots.

## 4. Materials and Methods

### 4.1. Materials

Polypropylene carbonate (PPC) was purchased from Sigma Aldrich (St. Louis, MO, USA). Polylactides of different grades, specifically 2003D (hereafter denoted as crystPLA) and 4060D (hereafter denoted as amorphPLA), were purchased from NatureWorks, Minnetonka, MN, USA. Chloroform was used as a solvent for polymers (Chempur, Piekary Śląskie, Poland). Potassium dihydrogen phosphate/di-sodium hydrogen phosphate (PBS with a pH value of 7.00 at 20 °C, Sigma Aldrich) was used as a medium for hydrolysis studies. Seeds of oat (*Avena sativa*) were purchased from Manufacturing and Trading Company Nieznanice, Malopolska Cultivation of Plants (Nieznanice, Poland). Seeds of common radish (*Raphanus sativus*) were purchased from PlantiCo (Stare Babice, Poland).

### 4.2. Preparation of Polymer Films

Polymer films were prepared by the solvent casting method. Appropriate amounts of polymers were weighed with an accuracy of 0.001 g on a RADWAG analytical balance (XA 110) and then dissolved in chloroform in a polymer/solvent ratio of 1:20 (*w*/*v*) and blended thoroughly using a magnetic stirrer. Homogenized solutions were poured out on Teflon dishes and left to preliminary evaporate chloroform. Afterwards, Teflon dishes were placed in a vacuum dryer (40 °C, 10 mBar) and left for a few days to remove residues of solvent. Blends of PLA/PPC were in the following ratios: 75/25, 50/50, and 25/75. Polymeric circle-shaped samples (15 mm in diameter, thickness ~100 µm) were cut from the obtained films and used for degradation studies in soil and PBS.

### 4.3. Soil Burial Test

The granulometric composition of the soil is otherwise known as the soil texture. It depends on the particle size of the mineral matter in the soil. Soil mineral particles are separated into three particle-size fractions: sand (>50 μm), silt (2–50 μm), and clay (<2 μm)—classification in accordance with the Soil Science Society of Poland. The soil used in this study meets the criteria of the OECD 208 guidelines [69]. According to this standard, tests were carried out in sandy soil with the following parameters: granulometric composition of the soil (79% of sand, 19% of silty, and clay, organic carbon content of approx. 2%) and pH (KCl) = 7.5.

In total, 250 g of soil was placed in polypropylene pots, and four disks of each blend multiplied by the number of determined time periods were buried in a pot at 1 cm under the soil surface. The pots were placed in a phytotron at a constant temperature of 22 ± 2 °C and watered every two days with the same amount of water to maintain the same level of humidity in the soil during the entire experiment duration. The degradation test was carried out for 24 months. Samples were removed after a specified period of time (3, 9, 18, and 24 months), thoroughly washed with distilled water, and dried to determine their rate of degradation by measuring weight loss, blend composition, surface erosion, and molecular changes in the samples.

### 4.4. Incubation in PBS

The same samples of polymer (described in Section 4.3) were immersed in PBS solution (glass bottle, 10 mL volume) and placed at 37 °C in an incubator shaker (IKA KS 4000i control, Königswinter, Germany) with a controlled temperature. Samples were removed after a specified period of time: 1, 3, 6, and 12 months, thoroughly washed with distilled water, and dried to determine their rate of degradation by measuring the same parameters as described in Section 4.3. After removing the samples, the pH of the medium was determined using the HI 9318 pH meter (Hanna Instruments, Villafranca padovana, Italy).

### 4.5. Determination of the Weight Loss

All discs were weighted before placing them into soil and PBS, and after a specified period of time, they were weighed using a RADWAG analytical balance with an accuracy of 0.0001 g.

### 4.6. pH Changes

The pH of the soil during the degradation process was determined in a suspension of soil and distilled water in a volume ratio of 1:5 using a pH meter. The pH of the PBS during the degradation process was determined directly in the solution.

### 4.7. Gel Permeation Chromatography (GPC)

The mass average molar mass (Mw), number average molar mass (Mn), and polydispersity index (*Ð* = Mw/Mn) of the tested samples were determined from peaks corresponding to the polymer fraction according to the absolute calibration method by GPC using a Waters HPLC system equipped with a Waters model e2695 and a Waters model 2414 differential refractometer (Waters Corporation, Milford, MA, USA). The samples were dissolved in THF (2–3 mg/mL), stabilized with BHT (240 mg/L), and filtered out using a syringe PTFE filter (pore size of 0.45 μm). The separation was carried out on a series of gel-mixed bed columns (Polymer Laboratories Ltd., Shropshire, UK) as follows: 1 × PLgel-Mixed-A bed column (300 × 7.5 mm, 20 μm), 1 × PLgel-Mixed-B bed column (300 × 7.5 mm, 10 μm), and 1 × PLgel-Mixed-D bed column (300 × 7.5 mm, 5 μm); the temperature of the mobile phase was 40 °C, its flow rate was set to 1.0 mL/min, and the injected volume equaled 100 μL. All data processing was carried out using Empower 3 software (version FR4).

### 4.8. Proton Nuclear Magnetic Resonance (^1^H NMR) Spectroscopy

Samples were dissolved in deuterated chloroform (CDCl_3_) (5 mg/mL) at room temperature. NMR spectra were recorded on an ECZ400R/S3 spectrometer (JEOL, Tokyo, Japan) operating at 400 MHz, equipped with a JASTEC 400/54 magnet and a ROAYAL HFX probe. ^1^H chemical shifts were related to the solvent signal. Spectra were processed using MNova 6 (Mestrelab Resources, Escondido, CA, USA).

The compositions of polymer blends were calculated based on the intensity of signals assigned to the proton of -CH- groups of PLA and PPC with the following equation:

(1) $$S_{\mathrm{PLA}}=\frac{J\cdot M_{\mathrm{PLA}}}{J\cdot M_{\mathrm{PLA}}+P\cdot M_{\mathrm{PPC}}}\cdot 100$$

where *J* is the signal intensity coming from the -CH- group of PLA; *P* is the intensity of the signal coming from the -CH- group of PPC; *M*_PLA_ is the molar mass of the LA monomer unit (72 g/mol); and *M*_PPC_ is the molar mass of the PC monomer unit (92 g/mol).

### 4.9. Differential Scanning Calorimetry (DSC)

DSC analysis was performed to assess crystallinity and thermal transitions in the PLA/PPC samples at various stages of the bio-degradation process. The data were collected using the DSC 1 STAR System (Mettler-Toledo, Switzerland) operating in the temperature range from −30 to 200 °C with a heating/cooling rate of 10 °C/min under a nitrogen purge of 40 mL/min. First, the thermal history of the samples was quenched, and then the DSC trace from the second heating scan was used to determine the glass transition temperature (*T*_g_), the cold crystallization temperature (*T*_cc_), the enthalpy of cold crystallization (Δ*H*_cc_), the melting temperature ™, and the enthalpy of melting (Δ*H_m_*). The degree of crystallinity (*χ_C_*) was calculated following the formula:

(2) $$\chi_{C}=\frac{\Delta H}{\Delta H_{m}^{0}\times W_{PLA}}\times 100\%$$

where Δ*H* equals (Δ*H_m_* − Δ*H*_cc_), Δ*H_m_* is the melting enthalpy of 100% crystalline PLA (93.0 J/g), and *W*_PLA_ is the weight fraction of the PLA [70]. This calculation was made assuming that PPC is an amorphous polymer [71].

### 4.10. Surface Erosion of Polymer Films

Surface evaluation of the polymeric films, after a specified period of their incubation time in each media, was conducted by the Tescan model VEGA 3SBU scanning electron microscope (SEM) (Tescan Orsay Holding, Brno, Czech Republic). Surface measurement was conducted using an accelerating voltage which ranged between 1 and 3 kV. Scan analysis of the surface was conducted at a low vacuum using the secondary electron mode, and to avoid samples damage and make measurements more reliable, microspheres were not coated with a conductive layer.

### 4.11. Plant Growth Test of PPC

To assess the phytotoxicity of PPC, a phytotron was utilized that met the standards outlined in the OECD 208 Guideline for Terrestrial Plants Growth Test. Two plants, oat (*A. sativa*) and radish (*R. sativus*), were chosen to represent monocotyledonous and dicotyledonous plants, respectively. The soil utilized for the phytotoxicity examination was the same as that utilized for the soil burial test (Section 4.3). Evaluation of plant growth was conducted in polypropylene (PP) pots (diameter of 90 mm and volume of 250 cm^3^), which were filled with either control soil or soil with sand (200 + 50 g of each, respectively) blended with the PPC. In detail, PPC was dissolved in chloroform, added to 50 g of sand, and mixed thoroughly. And it was then left until the solvent totally evaporated. Then, sand homogenized with dried PPC was added to 200 g of soil and mixed thoroughly with a mechanical stirrer. The polymer was added at varying concentrations of 250, 500, 750, and 1000 mg of PPC per kg of dry weight of soil. To ensure accuracy, each concentration was repeated three times, with three pots dedicated to oat and common radish each. For each plant, twenty seeds were planted beneath the soil surface from the same origin. The seedlings were grown under closely monitored conditions in the phytotron for fourteen days. Proper conditions were maintained to guarantee optimal plant growth and development, including a constant humidity level of 70% of the field water capacity, a temperature of 22 ± 2 °C, and a constant light intensity of around 7000 lux, regulated automatically in a 16 h day and 8 h night system. The phytotoxicity test introduced in this study is a common method used in the evaluation of toxicological endpoints such as biomass production, seed germination percentages, chlorophyll and carotenoid content, as well as seedling and root growth.

One-way analysis of variance (ANOVA) has been used for the comparison of obtained results related to phytotoxicity. Tukey’s test with *p* < 0.05 was used to determine the significance of the reported differences. The data presented in tables and figures are expressed as the mean with standard deviation obtained from 3 measurement replicates.

The effective concentration EC_50_ for the fresh matter of plants was calculated using GraphPad Prism software (Version 7, GraphPad Software, Inc., La Jolla, CA, USA). The dry weights (DWs) of the tested plants were measured after drying at 105 °C until the constant weight was reached, according to:

(3) $$DW=\frac{W_{D}}{W_{F}}\left(g/g\;offreshweight\right)$$

where *W*_D_ is the weight of the plant after drying and *W*_F_ is the weight of the fresh plant before drying.

In order to assess the effects of the PPC, the plants grown in soil were subjected to a visual examination. Digital photography was utilized in this process, and the resulting images were scrutinized for any indications of harm to the seedlings that were tested. This included the analysis of growth inhibition, as well as the identification of instances of chlorosis and necrosis.

To determine the photosynthetic pigment content, the method outlined by Oren et al. [72] was followed. Fresh leaves weighing 200 mg were homogenized using 20 mL of 80% acetone in a cooled mortar and then subjected to centrifugation (Model MPW 212, 4000 RPM through 10 min). The absorbance at wavelengths 470, 647, and 664 nm was utilized to calculate the chlorophyll a and chlorophyll b content (also referred to as total chlorophyll) and the carotenoid content. The final results were expressed as mg/g of the dry weight of the plants.

## 5. Conclusions

Our research demonstrates that PPC is a non-toxic, environmentally friendly polymer for edible plants, and with this respect, it can be successfully used in agriculture. In the case of PLA/PPC blends, the obtained results showed that only several percent of the examined blends underwent biodegradation within 24 months, and their vast majority was left in the soil. These outcomes are highly relevant for further compositional optimization of PPC-based formulations using other biodegradable and compatible polymers to develop films with a degradation time of around 6–8 months, i.e., plant cultivation time. We postulate that the incorporation of “green” or “eco” additives, such as plasticizers, could further facilitate the enzymatic degradation of PPC-based blends. Also, blending PPC with natural polymers such as PHB or starch could enhance enzymatic degradability in soil. In this study, PLA was used since it is one of the most known and used biodegradable polymers in many industrial branches (mostly due to its mechanical properties similar to those of PE or PET). The obtained results thus determine directions for agriculturalists to further investigate PPC-based blends for agricultural applications, including not only mulching films but also a wide range of controlled release formulations (CRFs) of agrochemicals with a time release no longer than the cultivation of plants.

The following conclusions can be drawn from the work:

(i) The degradation rate of the PLA/PPC blends was dependent on the ratio of both components regardless of the degradation medium; however, their time degradation was not satisfied from the mulching film application point of view;
(ii) PPC demonstrated the highest degradation rate compared to all the other tested samples, and its presence in the blend facilitated the degradation of PLA/PPC blends. For this reason, PPC is a highly promising material for further searching for more enzymatically degraded compatible blends;
(iii) Blends consisting of amorphous PLA and PPC demonstrated a faster degradation rate compared to their analogous blends based on the PLA grade that has the ability to crystalize;
(iv) The phytotoxicity test of PPC did not show any harmful effect of this polymer on monocotyledonous and dicotyledonous plants; however, the highest concentration slightly inhibited the development of oat roots, which requires attention and further research. In this respect, this polymer can be successfully used for environmental protection and agricultural purposes, including mulching films and CRFs of agrochemicals (pesticides, fertilizers).

## Figures and Tables

**Table 1:** GPC data for polymer samples incubated in soil.

Incubation in Soil															
Samples	Before Incubation			3 Months			9 Months			18 Months			24 Months		
	Mw [g/mol]	Mn [g/mol]	*Đ* [−]	Mw [%]	Mn [%]	*Đ* [−]	Mw [%]	Mn [%]	*Đ* [−]	Mw [%]	Mn [%]	*Đ* [−]	Mw [%]	Mn [%]	*Đ* [−]
PPC	211,200	40,000	5.28	99.0	103.0	5.09	88.8	81.5	5.76	93.2	85.5	5.75	69.3	80.8	4.54
crystPLA	249,200	125,500	1.99	95.5	98.7	1.91	100.4	128.6	1.55	89.5	120.5	1.47	46.8	50.9	1.82
amorphPLA	173,700	91,500	1.90	92.8	92.0	1.91	92.6	120.8	1.46	85.7	116.1	1.40	43.1	42.8	1.90
crystPLA/PPC 75/25	240,300	90,500	2.66	94.9	96.4	2.62	80.0	36.4	5.87	97.1	114.4	2.26	59.3	76.8	2.05
crystPLA/PPC 50/50	231,400	65,500	3.53	96.8	97.7	3.50	88.0	55.7	5.58	87.3	59.1	5.21	67.4	103.5	2.30
crystPLA/PPC 25/75	223,000	52,000	4.29	100.2	102.1	4.20	97.7	65.6	6.40	95.3	49.6	5.95	78.7	142.5	2.37
amorphPLA/PPC 75/25	185,700	78,400	2.37	92.1	93.6	2.33	102.8	71.9	3.39	106.1	111.4	2.26	49.2	50.9	2.29
amorphPLA/PPC 50/50	195,400	62,100	3.15	98.2	97.8	3.16	100.3	53.0	5.95	102.8	77.8	4.16	69.1	85.5	2.54
amorphPLA/PPC 25/75	206,300	48,200	4.28	96.8	95.2	4.35	99.1	65.6	6.47	99.9	68.0	6.25	74.6	119.3	2.68

**Table 2:** GPC data for polymer samples incubated in PBS.

Incubation in PBS															
Samples	Before Incubation			1 Month			3 Months			6 Months			12 Months		
	Mw [g/mol]	Mn [g/mol]	*Đ* [−]	Mw [%]	Mn [%]	*Đ* [−]	Mw [%]	Mn [%]	*Đ* [−]	Mw [%]	Mn [%]	*Đ* [−]	Mw [%]	Mn [%]	*Đ* [−]
PPC	211,200	40,000	5.28	83.4	81.0	5.43	83.4	72.0	6.11	84.1	81.8	5.43	62.6	100.8	3.28
crystPLA	249,200	125,500	1.99	92.5	124.9	1.47	69.7	78.2	1.77	53.0	54.1	1.95	4.4	4.9	1.76
amorphPLA	173,700	91,500	1.90	90.7	77.7	2.22	66.0	76.6	1.64	42.2	50.5	1.59	N/D*	N/D*	N/D*
crystPLA/PPC 75/25	240,300	90,500	2.66	93.6	72.7	3.42	88.8	91.4	2.58	72.5	67.5	2.85	24.0	21.4	3.02
crystPLA/PPC 50/50	231,400	65,500	3.53	104.8	97.1	3.81	92.8	74.1	4.43	88.8	84.7	3.71	46.3	57.4	2.85
crystPLA/PPC 25/75	223,000	52,000	4.29	99.5	85.8	4.97	96.6	87.3	4.75	93.3	84.4	4.73	62.6	92.7	2.90
amorphPLA/PPC 75/25	185,700	78,400	2.37	106.7	56.5	4.47	95.0	66.8	3.37	88.2	62.0	3.37	21.1	10.7	4.65
amorphPLA/PPC 50/50	195,400	62,100	3.15	109.4	69.9	4.92	105.2	76.5	4.33	102.1	73.4	4.37	51.6	21.1	7.70
amorphPLA/PPC 25/75	206,300	48,200	4.28	92.3	59.9	6.59	93.3	68.3	5.84	95.3	80.3	5.08	60.7	57.9	4.50

N/D*—peak was not detected.

**Table 3:** PLA/PPC—weight ratio (NMR) (-CH-/-CH-).

Incubation in Soil					
Sample	Before Incubation	3 Months	9 Months	18 Months	24 Months
crystPLA/PPC 75/25	79/21	78/22	83/17	84/16	78/22
crystPLA/PPC 50/50	56/44	56/44	60/40	62/38	57/43
crystPLA/PPC 25/75	31/69	31/69	29/71	30/70	33/67
amorphPLA/PPC 75/25	79/21	79/21	78/22	77/23	80/20
amorphPLA/PPC 50/50	56/44	56/44	55/45	54/46	56/44
amorphPLA/PPC 25/75	31/69	31/69	29/71	19/81	33/67
Incubation in PBS					
Sample	Before incubation	1 month	3 months	6 months	12 months
crystPLA/PPC 75/25	79/21	78/22	79/21	79/21	77/23
crystPLA/PPC 50/50	56/44	57/43	56/44	59/41	55/45
crystPLA/PPC 25/75	31/69	31/69	33/67	34/66	39/61
amorphPLA/PPC 75/25	79/21	79/21	81/19	80/20	76/24
amorphPLA/PPC 50/50	56/44	56/44	57/43	56/44	54/46
amorphPLA/PPC 25/75	31/69	30/70	29/71	31/69	29/71

**Table 4:** DSC data for the PLA/PPC blends (incubation in soil) taken after erasing their thermal history (second heating scan).

	$T_{g}^{PLA}$ (°C)	$T_{g}^{PPC}$ (°C)	*T*_cc_ (°C)	Δ*H*_cc_ (J/g)	*T*_m_ (°C)	Δ*H*_m_ (J/g)	*χ_C_* (%)
	Before Incubation						
PPC	N/D	11.8	N/D	N/D	N/D	N/D	N/D
crystPLA	44.6	N/D	104.6	22.6	139.8 144.4	−25.0	2.58
amorphPLA	38.5	N/D	N/D	N/D	N/D	N/D	N/D
crystPLA/PPC 75/25	38.6	19.1	103.8	19.9	137.2 143.4	−21.1	1.79
crystPLA/PPC 50/50	35.5	14.3	101.6	13.7	135.5 143.0	−14.9	2.58
crystPLA/PPC 25/75	36.8	17.1	101.8	7.41	137.6 144.6	−8.48	4.60
amorphPLA/PPC 75/25	35.0	16.1	N/D	N/D	N/D	N/D	N/D
amorphPLA/PPC 50/50	32.3	15.1	N/D	N/D	N/D	N/D	N/D
amorphPLA/PPC 25/75	31.9	14.6	N/D	N/D	N/D	N/D	N/D
3 months							
PPC	N/D	18.6	N/D	N/D	N/D	N/D	N/D
crystPLA	45.5	N/D	106.1	23.5	140.1 145.2	−27.5	4.39
amorphPLA	41.5	N/D	N/D	N/D	N/D	N/D	N/D
crystPLA/PPC 75/25	40.4	19.0	105.8	19.8	138.1 144.4	−20.2	0.66
crystPLA/PPC 50/50	41.0	19.1	105.5	15.4	139.7 145.2	−15.8	0.80
crystPLA/PPC 25/75	41.5	21.3	112.0	5.09	143.2	−5.70	2.62
amorphPLA/PPC 75/25	39.0	21.8	N/D	N/D	N/D	N/D	N/D
amorphPLA/PPC 50/50	37.2	18.1	N/D	N/D	N/D	N/D	N/D
amorphPLA/PPC 25/75	39.1	21.2	N/D	N/D	N/D	N/D	N/D
9 months							
PPC	N/D	20.9	N/D	N/D	N/D	N/D	N/D
crystPLA	43.8	N/D	104.0	27.1	138.5 146.2	−27.7	0.66
amorphPLA	41.9	N/D	N/D	N/D	N/D	N/D	N/D
crystPLA/PPC 75/25	42.4	21.0	107.6	22.4	139.8 146.0	−23.4	1.51
crystPLA/PPC 50/50	42.3	19.4	106.4	15.3	140.1 146.1	−16.0	1.38
crystPLA/PPC 25/75	44.1	26.3	112.6	4.50	144.2 149.9	−4.81	1.33
amorphPLA/PPC 75/25	41.4	23.1	N/D	N/D	N/D	N/D	N/D
amorphPLA/PPC 50/50	40.4	22.0	N/D	N/D	N/D	N/D	N/D
amorphPLA/PPC 25/75	44.4	27.9	N/D	N/D	N/D	N/D	N/D
18 months							
PPC	N/D	21.8	N/D	N/D	N/D	N/D	N/D
crystPLA	54.9	N/D	114.7	31.7	146.5	−31.7	0.00
amorphPLA	40.6	N/D	N/D	N/D	N/D	N/D	N/D
crystPLA/PPC 75/25	46.3	24.6	106.2	24.7	141.2 148.1	−24.7	0.00
crystPLA/PPC 50/50	40.3	20.1	103.4	18.6	138.9 146.8	−18.6	0.00
crystPLA/PPC 25/75	43.9	25.0	112.7	7.5	143.8 149.8	−7.5	0.00
amorphPLA/PPC 75/25	39.8	20.3	N/D	N/D	N/D	N/D	N/D
amorphPLA/PPC 50/50	40.6	22.6	N/D	N/D	N/D	N/D	N/D
amorphPLA/PPC 25/75	42.6	24.8	N/D	N/D	N/D	N/D	N/D
24 months							
PPC	N/D	22.8	N/D	N/D	N/D	N/D	N/D
crystPLA	45.2	N/D	101.1	38.6	136.9 146.6	−33.1	0.00
amorphPLA	42.1	N/D	N/D	N/D	N/D	N/D	N/D
crystPLA/PPC 75/25	42.1	20.4	104.3	27.60	137.8 145.9	−25.18	0.00
crystPLA/PPC 50/50	41.9	20.3	102.1	19.25	138.5 146.6	−17.86	0.00
crystPLA/PPC 25/75	45.9	26.9	114.4	6.69	144.5 *	−6.34	0.00
amorphPLA/PPC 75/25	40.1	21.8	N/D	N/D	N/D	N/D	N/D
amorphPLA/PPC 50/50	39.7	21.8	N/D	N/D	N/D	N/D	N/D
amorphPLA/PPC 25/75	43.7	27.4	N/D	N/D	N/D	N/D	N/D

* The abbreviations for DSC thermal characteristics are defined in Section 4.9.

**Table 5:** Average changes (mean of three replicates) in the germination of oat (*A. sativa*) and radish (*R. sativus*) treated with PPC. The least significant difference for samples (LSD_S_) and concentration (LSD_C_) is given for each tested parameter.

PPC Concentration in Soil (mg/kg of Soil Dry Weight)	OAT		RADISH	
	Number of Emerged Seedlings	Germination %	Number of Emerged Seedlings	Germination %
Control	18	100	18	100
100	19	104	18	96
250	19	104	17	93
500	19	104	17	95
1000	19	104	17	93
	LSD_S_ = 1 LSD_C_ = 2		LSD_S_ = 1 LSD_C_ = 1	

**Table 6:** Average changes (mean of three replicates) in fresh, dry mass, root length, and shoot height of oat (*A. sativa*) and radish (*R. sativus*) treated with PPC (percentage data are expressed as a mean ± SD of three replicates for each concentration).

PPC Conc. (mg/kg of Soil)								
	Fresh Mass (g/pot)	Fresh Mass Related to Control [%]	Dry Weight (mg/g of Fresh Weight)	Dry Mass Related to Control [%]	Average Shoot Height [cm]	Average Shoot Height Related to Control [%]	Average Root Length [cm]	Average Root Length Related to Control [%]
	Oat							
0	2.34	100	0.2264	100	20.0	100	11.5	100
250	2.36	100.7 ± 5.3	0.2282	100.8 ± 4.1	19.6	98 ± 1.1	11.4	99.1 ± 2.2
500	2.30	98.4 ± 6.5	0.2190	96.7 ± 5.8	19.1	95.5 ± 0.9	10.9	94.8 ± 2.6
750	2.41	102.9 ± 3.7	0.2158	95.3 ± 2.6	19.5	97.5 ± 0.8	10.5	91.3 ± 3.4
1000	2.36	100.7 ± 2.2	0.1908	84.3 ± 7.4	19.2	96 ± 1.3	9.8	85.2 ± 1.9
	Radish							
0	2.42	100	0.1679	100	9.1	100	8.5	100
250	2.94	121.4 ± 3.7	0.2005	119.4 ± 6.0	8.9	97.8 ± 1.6	8.8	103.5 ± 1.5
500	2.89	119.6 ± 12.4	0.2012	119.8 ± 14.5	9.2	101.1 ± 3.3	8.8	103.5 ± 1.8
750	3.17	131.3 ± 10.4	0.2151	128.1 ± 20.0	8.8	96.7 ± 1.2	8.6	101.2 ± 2.5
1000	3.10	128.2 ± 5.4	0.2186	130.0 ± 4.9	8.8	96.7 ± 1.5	8.4	98.8 ± 1.4

## Data Availability

Most of the data were included in the publication. The other source data presented in this study are available on request from the corresponding author.

## Supplementary Materials

The following supporting information can be downloaded at: https://www.mdpi.com/article/10.3390/ijms25010653/s1.
