# Supplementary material for: Degradation of Polylactic Acid/Polypropylene Carbonate Films in Soil and Phosphate Buffer and Their Potential Usefulness in Agriculture and Agrochemistry
Source: Int J Mol Sci. 2024 Jan 4;25(1):653. doi: 10.3390/ijms25010653 (PMC10779558; doi:10.3390/ijms25010653)
Supplement: Supplementary file 1 [file ijms-25-00653-s001.zip › ijms-2789719-supplementary.pdf]

Degradation of Polylactic Acid/Polypropylene Carbonate Films in Soil and Phosphate Buffer and Their Potential Usefulness in Agriculture and Agrochemistry

Izabela Szymanek[1], Martin Cvek[2], Diana Rogacz[1], Arkadiusz Żarski[1], Kamila Lewicka[1], Vladimir Sedlarik[2], Piotr Rychter [1]*

[1] Faculty of Science and Technology, Jan Dlugosz University in Czestochowa, 13/15 Armii Krajowej Av., 42-200 Czestochowa, Poland; izabela.szymanek@doktorant.ujd.edu.pl (IS), d.rogacz@ujd.edu.pl (DR), arkadiusz.zarski@ujd.edu.pl (AŻ), k.lewicka@ujd.edu.pl (KL), p.rychter@ujd.edu.pl (PR)
[2] Centre of Polymer Systems, University Institute, Tomas Bata University in Zlín, Trida T. Bati 5678, 760 01, Zlín, Czech Republic ; cvek@utb.cz (MC), sedlarik@utb.cz (VS)
* Correspondence: p.rychter@ujd.edu.pl (PR)

SUPPLEMENTARY DATA

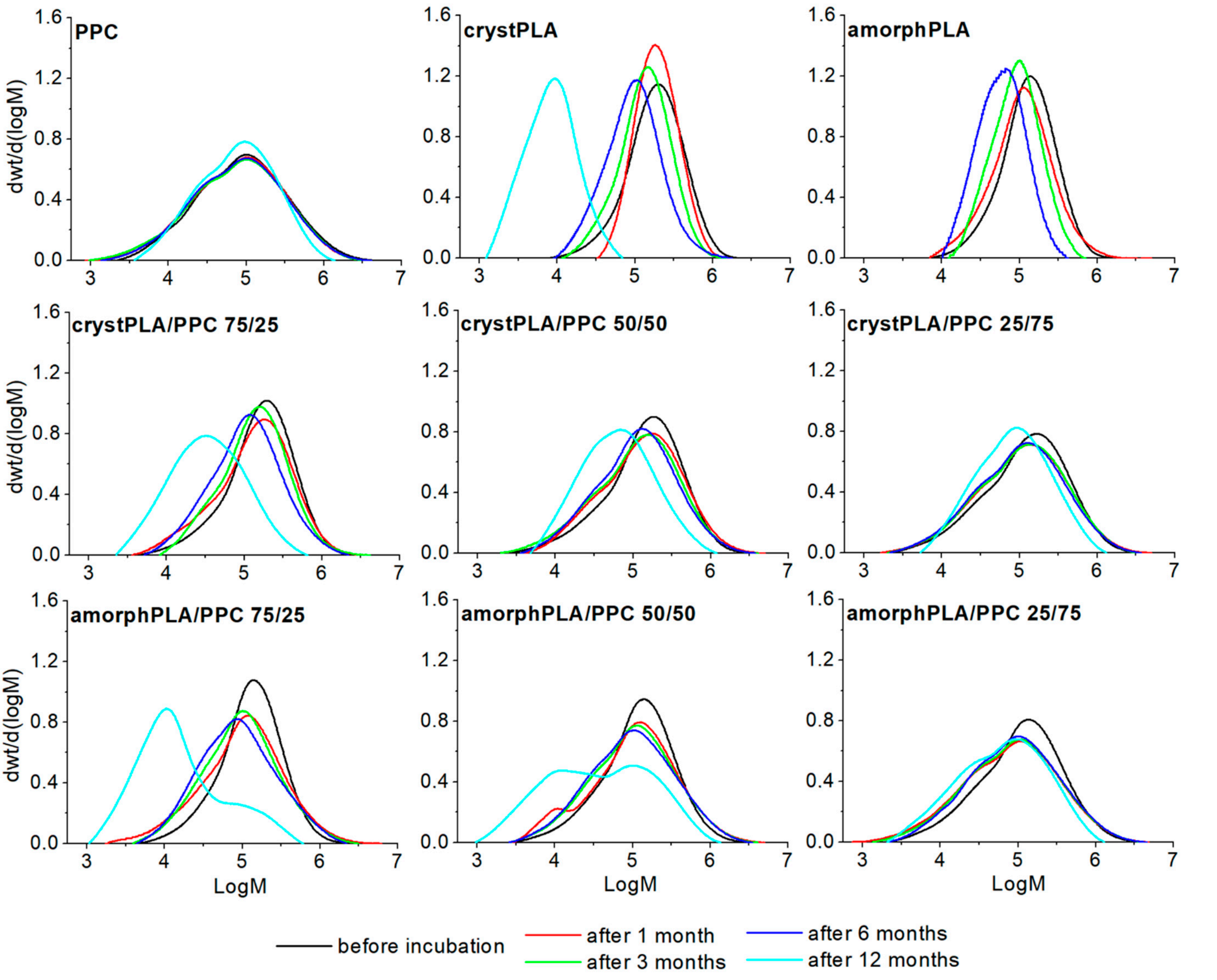

Figure S1. GPC elugrams of samples after incubation in PBS

Table S1. DSC data for the PLA/PPC blends (incubation in PBS) taken after erasing their thermal history (second heating scan).

	T_g^{PLA}(°C)	T_g^{PPC}(°C)	T_{cc} (°C)	ΔH_{cc} (J/g)	T_m (°C)	ΔH_m (J/g)	χ_C (%)
				**Before incubation**			
PPC	N/D	11.8	N/D	N/D	N/D	N/D	N/D
crystPLA	44.6	N/D	104.6	22.6	139.8<br>144.4	-25.0	2.58
amorphPLA	38.5	N/D	N/D	N/D	N/D	N/D	N/D
crystPLA/PPC 75/25	38.6	19.1	103.8	19.9	137.2<br>143.4	-21.1	1.79
crystPLA/PPC 50/50	35.5	14.3	101.6	13.7	135.5<br>143.0	-14.9	2.58
crystPLA/PPC 25/75	36.8	17.1	101.8	7.41	137.6<br>144.6	-8.48	4.60
amorphPLA/PPC 75/25	35.0	16.1	N/D	N/D	N/D	N/D	N/D
amorphPLA/PPC 50/50	32.3	15.1	N/D	N/D	N/D	N/D	N/D
amorphPLA/PPC 25/75	31.9	14.6	N/D	N/D	N/D	N/D	N/D
				**1 month**			
PPC	N/D	22.3	N/D	N/D	N/D	N/D	N/D
crystPLA	47.3	N/D	103.2	34.1	139.9<br>149.0	-34.2	0.15
amorphPLA	49.5	N/D	N/D	N/D	N/D	N/D	N/D
crystPLA/PPC 75/25	47.6	25.9	108.3	24.9	142.3<br>149.3	-26.2	1.79
crystPLA/PPC 50/50	40.2	20.3	106.1	17.1	140.4<br>147.9	-18.3	2.49
crystPLA/PPC 25/75	45.1	25.1	111.5	6.52	144.2	-6.90	1.63
amorphPLA/PPC 75/25	42.1	24.0	N/D	N/D	N/D	N/D	N/D
amorphPLA/PPC 50/50	40.8	22.8	N/D	N/D	N/D	N/D	N/D
amorphPLA/PPC 25/75	42.9	26.0	N/D	N/D	N/D	N/D	N/D
				**3 months**			
PPC	N/D	25.3	N/D	N/D	N/D	N/D	N/D
crystPLA	48.6	N/D	102.8	38.0	140.2<br>149.5	-38.5	0.54
amorphPLA	50.3	N/D	N/D	N/D	N/D	N/D	N/D
crystPLA/PPC 75/25	46.4	25.0	108.1	29.4	141.7<br>149.7	-29.4	0.00
crystPLA/PPC 50/50	48.6	27.3	108.9	16.0	143.4<br>150.0	-18.60	5.59
crystPLA/PPC 25/75	47.2	28.3	115.2	5.89	145.7*	-6.64	3.23
amorphPLA/PPC 75/25	44.1	26.4	N/D	N/D	N/D	N/D	N/D
amorphPLA/PPC 50/50	43.4	25.8	N/D	N/D	N/D	N/D	N/D
amorphPLA/PPC 25/75	44.1	27.5	N/D	N/D	N/D	N/D	N/D
				**6 months**			
PPC	N/D	25.1	N/D	N/D	N/D	N/D	N/D
crystPLA	48.6	N/D	109.2	41.4	142.3<br>150.6	-41.4	0.00
amorphPLA	49.2	N/D	N/D	N/D	N/D	N/D	N/D
crystPLA/PPC 75/25	46.3	26.2	113.5	29.6	143.3<br>150.6	-29.6	0.00
crystPLA/PPC 50/50	46.1	25.3	108.8	19.2	143.0<br>150.5	-19.2	0.00
crystPLA/PPC 25/75	54.6	34.4	116.3	5.40	149.3*	-5.40	0.00
amorphPLA/PPC 75/25	45.1	28.7	N/D	N/D	N/D	N/D	N/D

amorphPLA/PPC 50/50	44.3	28.1	N/D	N/D	N/D	N/D	N/D
amorphPLA/PPC 25/75	44.3	28.2	N/D	N/D	N/D	N/D	N/D
				**12 months**			
PPC	N/D	26.9	N/D	N/D	N/D	N/D	N/D
crystPLA	39.1	N/D	95.5	45.8	127.1<br>138.6	-42.3	0.00
amorphPLA	26.8	N/D	N/D	N/D	N/D	N/D	N/D
crystPLA/PPC 75/25	43.3	26.1	95.8	33.5	137.8<br>146.7	-32.4	0.00
crystPLA/PPC 50/50	42.0	26.9	96.5	20.7	138.5<br>147.8	-20.7	0.00
crystPLA/PPC 25/75	49.7	31.4	N/D	N/D	149.9*<br>152.2	-7.6	0.00
amorphPLA/PPC 75/25	44.7	31.7	N/D	N/D	N/D	N/D	N/D
amorphPLA/PPC 50/50	44.5	31.9	N/D	N/D	N/D	N/D	N/D
amorphPLA/PPC 25/75	46.0	33.0	N/D	N/D	N/D	N/D	N/D

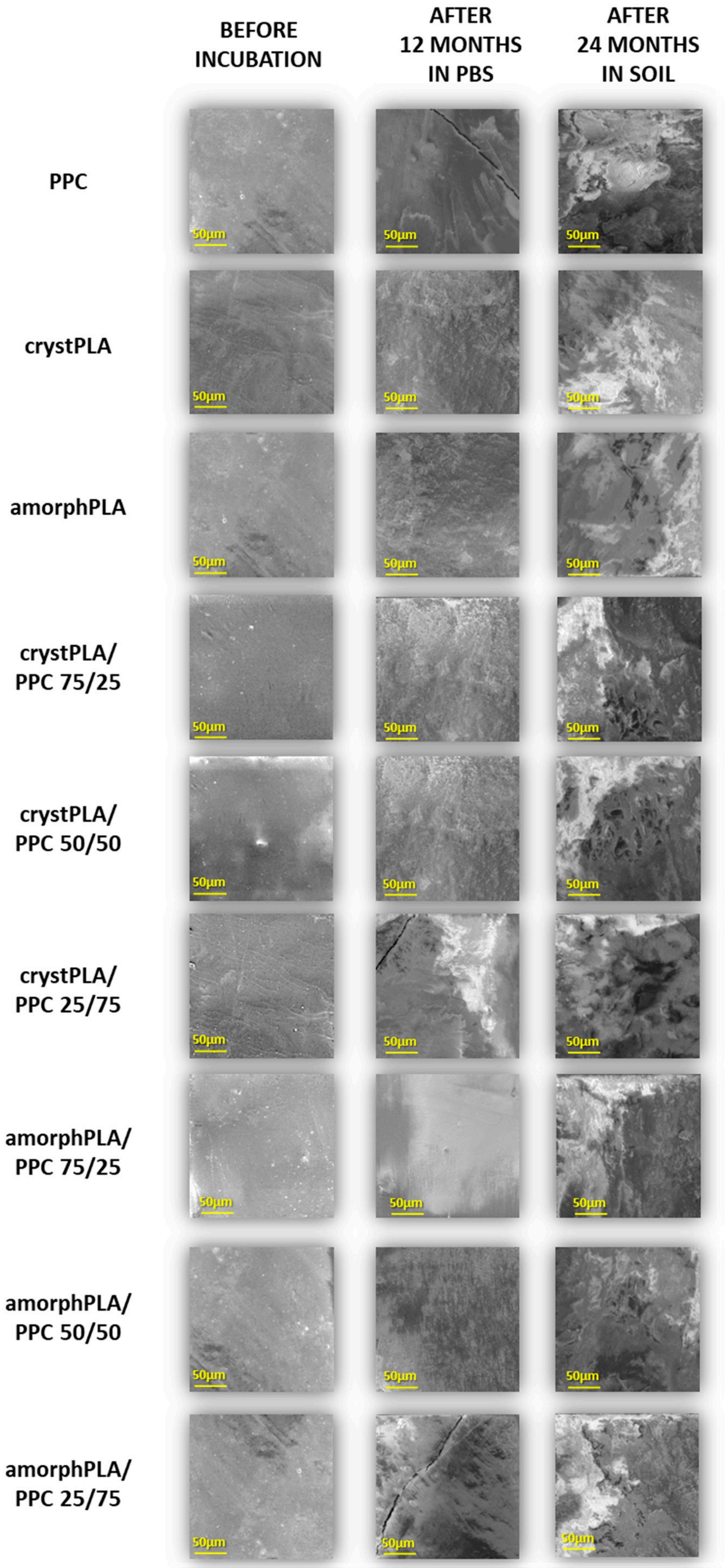

Figure S2. A list of the SEM micrographs of the surfaces of incubated foils before and during incubation.
